# Far away from the lamppost

**DOI:** 10.1371/journal.pbio.3000067

**Published:** 2018-12-11

**Authors:** Tudor I. Oprea, Lily Jan, Gary L. Johnson, Bryan L. Roth, Avi Ma’ayan, Stephan Schürer, Brian K. Shoichet, Larry A. Sklar, Michael T. McManus

**Affiliations:** 1 Department of Internal Medicine, Comprehensive Cancer Center, University of New Mexico School of Medicine, Albuquerque New Mexico, United States of America; 2 Department of Physiology, University of California–San Francisco, San Francisco, California, United States of America; 3 Department of Pharmacology, Lineberger Comprehensive Cancer Center, University of North Carolina School of Medicine, Chapel Hill, North Carolina, United States of America; 4 Department of Pharmacology, University of North Carolina School of Medicine, Chapel Hill, North Carolina, United States of America; 5 Department of Pharmacological Sciences, Icahn School of Medicine at Mount Sinai, New York, New York, United States of America; 6 Department of Pharmacology, Miller School of Medicine, Center for Computational Science, University of Miami, Miami, Florida, United States of America; 7 Department of Pharmaceutical Chemistry, University of California San Francisco, San Francisco, California, United States of America; 8 Department of Pathology, Comprehensive Cancer Center, University of New Mexico School of Medicine, Albuquerque, New Mexico, United States of America; 9 Department of Microbiology and Immunology, UCSF Diabetes Center, University of California–San Francisco, San Francisco, California, United States of America

## Abstract

This Formal Comment responds to a recent Meta-Research Article by identifying initiatives that are already in place for funding risky exploratory research that illuminate mysteries of the dark genome.

One of the most exciting scientific accomplishments of this last century is the elucidation of the human genome sequence. This ambitious project cost hundreds of millions of dollars and was akin to sending humans to the moon. At the time, some wondered whether we are probing where no one should. They argued that successfully deciphering the veritable blueprint of life might forever amputate the intrinsic mysteries of humanity. Yet decades later, it is quite clear that scientists are still far from understanding the true depth and meaning of the 3.3 billion base pairs that make up the human genome.

An article published in *PLOS Biology* by Amaral and colleagues [1] reiterated some of the challenges associated with exploring this vast treasure trove of human DNA. Rather than systematically studying and assigning meaning to each gene in the human genome, the global research enterprise remains focused on a small proportion of the human genome that has already been studied intensively. This has left thousands of genes in an understudied situation, even though many of them share associations with human health and disease. We previously noted [2] that understudied proteins “are less likely to be the subject of scientific curiosity, which is a reflection of funding patterns and an overall lack of information and molecular probes.”

Recognizing this fundamental shortcoming, the National Institutes of Health (NIH) addressed this challenge by launching the Illuminating the Druggable Genome (IDG) program [2,3] in 2014, with the explicit goal of transforming basic science and drug discovery by shedding light on the “dark genome.” The IDG program, now in its fourth year, has begun the slow process of reversing human scientific habits, encouraging scientists to apply for funds specifically to interrogate the “dark genome.” Currently, the IDG aggregates knowledge across all proteins coded by the human genome, assesses the knowledge state of each protein, and focuses its experimental efforts on generating new technologies and new data around the darkest of druggable genes, specifically those that have the most immediate prospects of improving human health by means of novel therapies. With success, we hope this effort will demonstrate the feasibility and value of illuminating understudied proteins, which can then be applied to additional protein families.

In addition to the IDG, there are other significant ongoing efforts by the NIH and other research foundations, including those inside and outside the United States. These include the Structural Genomics Consortium (SGC), which supports unbiased and systematic research, helping to deduce the three-dimensional structures of proteins—an important goal for understanding basic biology and developing new medicines. In addition, there is the Knockout Mouse Project (KOMP) and the International Mouse Phenotype Consortium (IMPC) [4,5], an international consortium whose goal is to generate and characterize a knockout mouse strain for every gene. The IMPC has partnered with the IDG to identify “dark protein coding genes” and prioritized the production of orthologous knockout strains. Indeed, the IMPC has already investigated 47% of the 500 genes that were reported to be understudied by the Amaral and colleagues article. Further, the Library of Integrated Network-based Cellular Signatures (LINCS) is helping to illuminate functional connectivity between well-studied and dark genes. These programs are helping to lead systematic efforts to assign functions to every gene in the genome and provide high-value fundamental data and resources. As we gain further insight into the biology of human health and disease, these programs can contribute to the development of new therapeutic targets. Instead of looking for keys under the lamppost, where the light shines, the IDG, SGC, IMPC, and many other programs make it possible to systematically study the “dark genome.”

We feel impelled to draw attention to extremely valuable data and a growing collection of community resources (Table 1). As the Amaral article did not include these types of data in their analyses, it underscores the challenges associated with awareness of a growing collection of data and resources that bear relevance to understanding the human genome. The fact that some of these programs have been producing reagents and experimental resources for over a decade suggests a larger problem with transparency and outreach. Some funded programs are systematically producing data and resources at a faster pace than researchers can be aware of them, outpacing the race to understand the rich complexity of human genome. Other programs stitch data elements from biomedical literature, NIH-funded grants, clinical trials and worldwide patents, in addition to combining evidence from genome-wide and mouse phenotype studies (Pharos, Harmonizome). These imbalances reflect the slow process of the research enterprise and pose a gentle reminder that the extraordinary accomplishment of the human genome–sequencing project is really the first step of a long and more arduous journey.

**Table 1:** Links to programs and resources mentioned in this article.

Programs	Link
International Mouse Phenotyping Consortium	https://www.mousephenotype.org
Illuminating the Druggable Genome Consortium	http://druggablegenome.net
Library of Integrated Network-based Cellular Signatures	http://lincsproject.org
Structural Genomics Consortium	https://www.thesgc.org
**Resources and databases**	
Pharos	https://pharos.nih.gov/idg/index
Harmonizome	http://amp.pharm.mssm.edu/Harmonizome

In the larger picture, it’s important to recall that research is anything but systematic and organized. Like bees to honey, scientists swarm to genes that promise a better understanding of what makes us human and how to cure disease. However, scientists are also guided by availability of research support, and as Amaral and colleagues pointed out, there are likely other systemic barriers to individual-level researchers embarking on study of these neglected proteins. Funded research is often granted to study those genes which have significant pre-existing knowledge that bolster specific hypotheses. To avoid this “catch 22” situation, the NIH and funding agencies in Europe, Canada, and elsewhere have established systematic programs dedicated to helping adjust the organic growth of the research enterprise by making data and reagents available to investigators. Although these programs are far from perfect and far from complete, we should continue the increasing trend of funding the “dark genome,” despite the risky and exploratory nature of exploratory research, accelerating science by shining light on what makes us human.

## Abbreviations

IDG: Illuminating the Druggable Genome
IMPC: International Mouse Phenotype Consortium
KOMP: Knockout Mouse Project
LINCS: Library of Integrated Network-based Cellular Signatures
NIH: National Institutes of Health
SGC: Structural Genomics Consortium
